# Assessment of Segmentary Hypertrophy of Future Remnant Liver after Liver Venous Deprivation: A Single-Center Study

**DOI:** 10.3390/cancers16111982

**Published:** 2024-05-23

**Authors:** Bader Al Taweel, Gianluca Cassese, Salah Khayat, Maurice Chazal, Francis Navarro, Boris Guiu, Fabrizio Panaro

**Affiliations:** 1 Department of Digestive Surgery and Liver Transplantation, Montpellier University Hospital, 34090 Montpellier, Francesalah.khayat@ch-perpignan.fr (S.K.);; 2 Department of Clinical Medicine and Surgery, Division of Minimally Invasive and Robotic HPB Surgery and Transplantation Service, University of Naples “Federico II”, 80131 Naples, Italy; gianluca.cassese@unina.it; 3 Department of Visceral and Digestive Surgery, Centre Hospitalier de Perpignan, 66000 Perpignan, France; 4 Department of General and Visceral Surgery, Centre Hospitalier Princesse Grace, 98000 Monaco, Monaco; maurice.chazal@chpg.fr; 5 Department of Diagnostic and Interventional Radiology, Montpellier University Hospital, 34090 Montpellier, France; b.guiu@chu-montpellier.fr; 6 Department of Surgery, Università del Piemonte Orientale, 15121 Alessandria, Italy

**Keywords:** liver venous deprivation, portal vein embolization, future liver remnant

## Abstract

**Simple Summary:**

Portal vein embolization, liver venous deprivation (PVE + right hepatic vein embolization) and extended liver venous deprivation (LVD + middle hepatic vein embolization) are three techniques used to induce hypertrophy of the remnant liver prior to a major hepatectomy. To date, the hypertrophy of the different liver segments has never been evaluated. We analyzed results from 44 patients (26 PVE, 10 LVD and 8 eLVD) and found that segments 1 and 2+3 had a greater degree of hypertrophy the more the embolization technique was advanced (eLVD > LVD > PVE). Segment 4’s hypertrophy did not seem to be affected by the embolization technique. These findings could help better understand liver hypertrophy and allow for a more personalized approach prior to surgery.

**Abstract:**

**Background**: Liver venous deprivation (LVD) is a recent radiological technique that has shown promising results on Future Remnant Liver (FRL) hypertrophy. The aim of this retrospective study is to compare the segmentary hypertrophy of the FRL after LVD and after portal vein embolization (PVE). **Methods**: Patients undergoing PVE or LVD between April 2015 and April 2020 were included. The segmentary volumes (seg 4, seg2+3 and seg1) were assessed before and after the radiological procedure. **Results**: Forty-four patients were included: 26 undergoing PVE, 10 LVD and 8 eLVD. Volume gain of both segment 1 and segments 2+3 was significantly higher after LVD and eLVD than after PVE (segment 1: 27.33 ± 35.37 after PVE vs. 38.73% ± 13.47 after LVD and 79.13% ± 41.23 after eLVD, *p* = 0.0080; segments 2+3: 40.73% ± 40.53 after PVE vs. 45.02% ± 21.53 after LVD and 85.49% ± 45.51 after eLVD, *p* = 0.0137), while this was not true for segment 4. FRL hypertrophy was confirmed to be higher after LVD and eLVD than after PVE (33.53% ± 21.22 vs. 68.63% ± 42.03 vs. 28.11% ± 28.33, respectively, *p* = 0.0280). **Conclusions**: LVD and eLVD may induce greater hypertrophy of segment 1 and segments 2+3 when compared to PVE.

## 1. Introduction

Surgery is the only curative treatment for hepatic tumors. In the last decades, hepato-biliary surgery has known a lot of technological progress, with great impact on both postoperative and survival outcomes. However, mortality after major hepatectomy varies between 3.1% and 4.5%, and severe complications occur in up to 20% of cases [1,2,3]. The major cause of mortality is post-hepatectomy liver failure (PHLF) [4,5]. It occurs in less than 3% of the cases, in patients without underlying liver disease, but is the first cause of death after major hepatectomy. PHLF is responsible for 18% of all postoperative deaths after major hepatectomy [3,5].

Different risk factors for PHLF have been investigated. The first is an underlying liver disease, such as cirrhosis. In this case, the PHLF rate can be as high as 20% after major hepatectomy [6,7]. The other factor causing PHLF is the future remnant liver volume (FRL) [8]. An FRL< 20–25% in patients without underlying liver diseases is a major factor of PHLF [9]. Many techniques have emerged to optimize the FRL and to induce hypertrophy [10]. 

The portal vein embolization is the current standard of care technique to induce FRL hypertrophy [11,12,13]. However, PVE does not always induce fast and sufficient hypertrophy, explaining that 20% of the patients will not be resected [12,14,15]. To try to solve this problem, associating liver partition and portal vein ligation for staged hepatectomy (ALPPS) has been developed, as well as many modifications to the original ALPPS technique, allowing to induce faster and higher hypertrophy, but it faces a much higher morbidity and mortality rate [16]. Another technique has emerged in the last ten years, the liver venous deprivation (LVD), which consists in PVE and hepatic vein embolization simultaneously [17]. A technical variant is the extended LVD (eLVD), where the median hepatic vein is also embolized. This strategy might generate a faster and greater FRL hypertrophy, and it may represent an alternative option, as these different strategies can be tailored case by case depending on the disease extension and the patient anatomy and condition [18]. 

Some preliminary studies focused on the differences between PVE and LVD/eLVD, the degree of hypertrophy, postoperative complications, delay between embolization and surgery, but to our knowledge, none of them have compared the segmentary volumetry and the distribution of hypertrophy in the FRL [19,20,21]. 

Our aim is to evaluate and compare the hypertrophy of the different segments of the FRL after PVE and after LVD in patients before major hepatectomy.

## 2. Methods

### 2.1. Study Design

This study is a mono-centric observational retrospective study. An informed consent was required before both the radiological embolization procedure and the surgical operation. This study was approved by the institutional review board and registered in clinicaltrials.gov (# IRB-MTP_2020_04_202000444, clinicaltrials.gov ID: NCT04370132), and the Declaration of Helsinki was respected.

### 2.2. Patient Selection

We included consecutive patients who underwent either PVE or LVD/eLVD before major hepatectomy as defined by the Brisbane classification in the Montpellier University Hospital between January 2015 and December 2020 [22].

Liver cirrhosis was an exclusion criterion.

### 2.3. Choice of PVE versus LVD/eLVD

The therapeutic strategy was discussed in the institutional multidisciplinary tumor board. The decision to perform a hypertrophy procedure before surgery was based on the expected FRL volume and/or the functional assessment based on Tc-99m mebrofenin scintigraphy. Indeed, in our center, liver growth strategies are considered when expected FRL is <25–30% in normal liver, <35–40% in case of an underlying liver disease (steatosis, neoadjuvant chemotherapies), <50% in case of cirrhosis, or when the extracting of Tc-99 mebrofenin of FRL is below 2.69%/min/m^2^ [23]. In patients with low volume and function FRL, LVD or eLVD was chosen. In patients with low-volume FRL and satisfactory function FRL or vice versa, PVE was performed. Moreover, the more volume FRL tended towards 20%, the more the board decided on LVD or eLVD instead of PVE. Finally, the board would also choose LVD or eLVD in cases of complex hepatectomies where the perioperative setting could modify the type of hepatectomy. The operation was planned starting from 3 weeks after the embolization, depending on the results of the volume analysis on the CT scan.

The decision for each patient reflected real-life practices and the complex choice-making during the multidisciplinary board, also given the novelty of the LVD procedure and the drawbacks of PVE.

### 2.4. Radiological Procedure 

During LVD, the right hepatic vein (and accessory right when present) was assessed under ultra-sonographic guidance, and a 0.018 inch microguidewire was inserted and left in place. Then, PVE was performed using right transhepatic access. After 3D portography, right portal vessels were embolized using a mixture of n-butyl cyanoacrylate and lipiodol (ratio 1:6). The microguidewire left in place in the hepatic vein was then used to insert a 7F- sheath in order to display an Amplatzer vascular plug II (75% oversizing).

Finally, all distal venous branches were embolized using a mixture of n-butyl cyanoacrylate and lipiodol.

In eLVD, the median hepatic vein is also embolized using transhepatic access.

### 2.5. Surgical Procedure 

Intra-operative ultrasonography was routinely performed. Pringle maneuver with intermittent clamping and right hepatic vein control were performed if necessary. The parenchymal dissection was carried out with a Cavitron ultrasound aspirator (CUSA) and/or harmonic scalpel and bipolar forceps. Hemostatic agents were used according to the surgeon’s decision.

### 2.6. Data and Volume Analysis

We collected the following data: age, sex, weight, type of cancer, number of nodules, sum of nodules diameters, preoperative chemotherapy, preoperative TACE, type of embolization, delay between embolization and surgery, volumetric assessments. The first volumetric measure was performed on the last CT scan or MRI available before the embolization procedure and constituted the reference volume. The second measure was performed on the last CT scan or MRI available before surgery. 

Liver volumes were calculated from manual reconstruction, and FRL-volume (FRL-V) was assessed using the formula described by Vauthey et al.: FRL-V = FRL-V/(eTLV − TV) × 100, where eTLV = –794.41 + 1267.28 × body surface area. To compare hypertrophy responses, the kinetic growth rate (KGR) was calculated as the percentage growth per day [degree of hypertrophy (DH) at the first post-procedural volume assessment (%)/elapsed interval from the radiological procedure (days)]. The degree of hypertrophy was calculated using the following formula (post − procedural FRL − V%) − (pre − procedural FRL − V%). Volumes were compared with the last imaging exam performed before surgery. In particular, we manually measured the following volumes: total liver, segment 4, segments 2+3 and segment 1. 

With the volume growth (VG) in milliliters, we calculated volume gain in percentage (VG-%) for each segment as the volume growth rate divided by preoperative volume, as a percentage.

Finally, we calculated the remnant liver volume-to-body-weight ratio.

### 2.7. Statistical Analysis 

Continuous data were expressed either in mean and standard deviation (SD) and then compared using a one-way ANOVA, or in median and interquartile range (IQR) and then compared using Kruskal–Wallis test, depending on the normality of data. 

For categorical data, the number and proportion were displayed. Qualitative variables were compared by the Chi square test or Fisher’s exact test when necessary. Variables were considered significant when the *p*-value was inferior or equal to 0.05.

Analyses were performed in SAS Enterprise 8.2.

## 3. Results

### 3.1. Patients’ Characteristics

**Figure 1:**
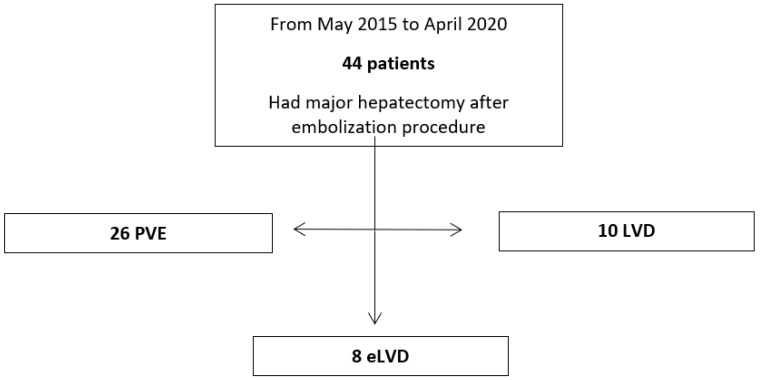
Flowchart: diagram of patient selection in case of major liver resection with high risk of post-hepatectomy liver failure; eLVD: extended liver deprivation (portal vein embolization + right and middle hepatic vein embolization); LVD: liver venous deprivation; PVE: portal vein embolization.

We included 44 patients in our study: 26 PVE, 10 LVD and 8 eLVD. Patients either had a right hepatectomy or an extended right hepatectomy (Figure 1).

There were no statistically significant differences in most preoperative patients’ characteristics (Table 1), except for the rate of preoperative chemotherapy (43% before PVE vs. 80% before LVD and 88 before eLVD) and the number of nodules (1 in the PVE group vs. 2.5 in the LVD group vs. 3.5 in the eLVD group).

The delay between embolization and surgery was not statistically significant: 27.4 days (SD 12.3), 23.2 days (SD 5.3), 25.4 days (SD 9.5), *p*-value 0.5590 between PVE, LVD and eLVD, respectively.

### 3.2. Volume Analysis

Results regarding VG are shown in Table 2. The VG of segments 1 and 2+3 were statistically significant between different groups (*p* = 0.0011 and 0.0108, respectively), whereas that of segment 4 was not (*p* = 0.8362). VG-% was better after eLVD for segments 2+3 and segment 1 (Table 3), too. Again, results for segment 4 were not statistically significant.

To avoid bias regarding time to surgery, we also calculated KGR (Table 4). The KGR of segment 1 was statistically significantly higher after eLVD/LVD than after PVE (*p* = 0.0019), while KGR of segments 2+3 and that of segment 4 were not.

Finally, the remnant liver volume-to-body-weight ratio showed a greater increase after eLVD (+0.357%) than LVD (+0.232%) and PVE (+0.240%), and the result was statistically significant (*p* = 0.049).

## 4. Discussion

Our results show that LVD and eLVD are associated with a higher VG and VG-% for segment 1 than PVE (LVD 11, eLVD 28.5 versus PVE 8.5, *p* = 0.0011) and segments 2+3 (LVD 112, eLVD 208 versus PVE 104, *p* = 0.0108) and a higher KGR for segment 1 (LVD 0.174, eLVD 0.272 versus PVE 0.106, *p* = 0.0019). KGR for segments 2+3 has a tendency toward statistical significance (LVD 1.891, eLVD 2.886 versus PVE 1.724, *p* = 0.1309). Both measurements showed no statistical difference between the three techniques regarding segment 4 (*p* = 0.8362 and 0.9407, respectively, for VG and KGR). The gain in the remnant liver volume-to-body-weight ratio was statistically significant and in favor of eLVD.

On a preliminary impression, these results may be seen as not directly clinically relevant. However, they may be very useful in a tailored approach for each patient, taking into account the type of hepatectomy needed and the anatomy of the patient. If a tumor requires a right hepatectomy and may require sacrifice of the fourth segment, we should straight away consider an eLVD, since our results suggest a greater hypertrophy of segments 1, 2 and 3 and no negative impact on segment 4.

PVE and subsequent innovative techniques such as LVD and eLVD have the same goal: to induce a quick and important hypertrophy of the remaining liver [9,10,24]. This hypertrophy is more important when embolization of the outflow system is also performed [21]. However, it is not always associated with an increase in liver function. LVD-induced volume hypertrophy was shown to be associated with a gain in function [25], which is not the case of other more effective hypertrophy procedures, such as Associating Liver Partition with Portal vein ligation for Staged hepatectomy (ALPPS) [26,27,28,29]. LVD also has the advantage of a lower morbidity and mortality when compared to ALPPS [20,30,31,32], which may pave the way to a wider use of LVD, saving ALPPS for specific situations, such as a salvage strategy [20,23].

We know that the portal flow is one important element to increase volume of the FRL; it stimulates the release of proliferative factors [8]. Increase in portal vein flow is in itself a predictive factor of hypertrophy [33,34]. PVE is now a standard method used widely to induce hypertrophy [35]. Nevertheless, even with PVE there is 15% up to 20% of drop out, mainly caused by disease progression or insufficient FRL hypertrophy [12,14]. Since 2016, Guiu et al. proposed the LVD technique to try and reduce the drop rate by generating greater and faster hypertrophy [17]. This technique allows to modulate the inflow and outflow, and rapid development of neo-collaterals between the portal and hepatic veins. Segment 4 is located on the border of the right and left liver and contains vascular communication with both the embolized liver and the FRL.

We initially thought that segment 4 would have a greater hypertrophy in case of LVD and eLVD [18]. Even though the results are not statistically significant, our series showed a better hypertrophy with PVE. Obstruction of part of segment 4 outflow might explain the lack of significant hypertrophy in eLVD, but the mechanisms behind the small hypertrophy of segment 4 after LVD are not immediately understandable. This has already been investigated in a small series using the living donor model [36].

It is interesting to note that previous studies about segmentary hypertrophy response after PVE have been previously published, while no papers tried to address this issue when dealing with LVD [37]. Hammond et al. reported a retrospective series of 60 patients, showing a greater DH of segments 2+3 after PVE with segment 4 embolization than PVE alone [38]. Such results are consistent with our findings, where segment 2+3 hypertrophy was greater after eLVD than after LVD alone (VG-% 85.49% vs. 45.02, respectively). In addition, segment 1 hypertrophy was also higher after eLVD than LVD alone (VG-% 79.13% vs. 38.73%, respectively), suggesting similar effects after segment 4 venous output occlusion. Finally, FRL volume gain was also higher after eLVD than LVD alone (68.63% vs. 33.53%, respectively).

The mean delay between LVD and completion of surgery may still appear high after LVD and eLVD (23.2 ± 5.3 days and 25.4 ± 9.5 days, respectively). However, during the introduction of the LVD technique, it has been regarded as a PVE-like technique: the longer the waiting time, the greater the FLR augmentation. Thus, it has been initially linked to a protocol aiming to wait about 4 weeks of interval before completion of surgery, similar to PVE, the current standard of care. Nonetheless, many previously published results showed a strong reduction in this delay [20,21]. Further studies and efforts should focus on interval time reduction when applying LVD procedures [20,21]. 

Our study presents several limitations. First, it is a retrospective study, with an inevitable risk of selection bias. There was no matching between the groups. However, there were no significant differences in population characteristics, except for the number of nodules and the rate of preoperative chemotherapy. Previous studies showed that FRL hypertrophy may be impaired by preoperative chemotherapy [39,40] even though most studies concluded that chemotherapy did not affect FRL hypertrophy [41,42,43,44,45]. In all cases, chemotherapy (more represented in our LVD and eLVD patients) would not increase FRL hypertrophy, reducing the risk of bias selection in our study. Secondly, we included all types of tumors. Patients with a Klatskin tumor may have an altered hypertrophy response due to cholestasis even though studies do not all confirm this matter [8,12,46,47,48,49]. Thirdly, since this is a retrospective study including patients undergoing major hepatectomy after LVD or PVE, the rate of eventual patients failing to undergo surgery after the radiological procedure was impossible to investigate. However, previous studies reported success rates as high as 100% [20]. Finally, the sample size was too small to draw a robust conclusion.

Future prospective studies with a larger sample size are needed to deeply evaluate segmentary volumes and functions to continue our understanding of the different mechanisms of liver hypertrophy, ideally leading to improved resection rates and improved postoperative outcomes.

## 5. Conclusions

In conclusion, LVD and eLVD may induce greater hypertrophy of segment 1 and segments 2+3 when compared to PVE, while such difference is not present for segment 4. Despite needing confirmation by further prospective studies, such results may guide a tailored surgical approach based on patients’ and tumor characteristics.

## Figures and Tables

**Table 1:** Preoperative patients’ characteristics. HCC: hepatocellular carcinoma; CCK: Cholangiocellular carcinoma; CRLM: colorectal liver metastases; PVE: portal vein embolization; LVD: liver venous deprivation; eLVD: extended LVD. Within the brackets, standard deviations or interquartile ranges. In bold, significant *p*-values.

Variable	PVE (26)	LVD (10)	eLVD (8)	*p*-Value
Age: mean in years (SD)	65.6 (9.5)	59.9 (9.9)	63.1 (7.3)	0.25
Sex				0.06
Female	12 (46%)	3 (30%)	0 (0%)	
Male	14 (54%)	7 (70%)	8 (100%)	
Type of cancer				0.45
HCC	6 (23%)	1 (10%)	0 (0%)	
CCK	7 (27%)	2 (20%)	1 (12%)	
CRLM	13 (50%)	7 (70%)	7 (88%)	
Preoperative chemotherapy				**0.03**
No	15 (57%)	2 (20%)	1 (12%)	
Yes	11 (43%)	8 (80%)	7 (88%)	
Preoperative TACE				0.46
No	22 (84%)	10 (100%)	8 (100%)	
Yes	4 (16%)	0 (0%)	0 (0%)	
Mean FRL volume (SD)	30% (7)	29% (5)	27% (7)	0.55
Median number of nodules (IQR)	1 (1)	2.5 (4)	3.5 (1.5)	**0.03**
Median sum of nodules diameters (IQR)	65 (68)	51.5 (23)	53 (57.5)	0.83

**Table 2:** Volume growth, in milliliters. PVE: portal vein embolization; LVD: liver venous deprivation; eLVD: extended LVD. Within the brackets, standard deviations.

Variable	PVE (n = 26)	LVD (n = 10)	eLVD (n = 8)	*p*-Value
VG—segment 1 (SD)	8.5 (13)	11 (19)	28.5 (14)	<0.01
VG—segments 2+3 (SD)	104 (115)	112 (25)	208 (82)	0.01
VG—segment 4 (SD)	31 (52)	34 (50)	53 (58)	0.84
VG—FRL (SD)	154 (158)	162 (188)	273 (168)	0.01
VGR—embolized liver (SD)	−132 (271)	−72 (295)	−60 (267)	0.93

**Table 3:** Volume gain in percentage. PVE: portal vein embolization; LVD: liver venous deprivation; eLVD: extended LVD. Within the brackets, standard deviations.

Variable	PVE (n = 26)	LVD (n = 10)	eLVD (n = 8)	*p*-Value
Volume gain—segment 1 (SD)	27.3 (35.4)	38.7 (13.5)	79.1 (41.2)	<0.01
Volume gain—segments 2+3 (SD)	40.7 (40.5)	45.0 (21.5)	85.4 (45.5)	0.01
Volume gain—segment 4 (SD)	16.5 (28.4)	17.5 (30.9)	22.6 (41.9)	0.79
Volume gain—FRL (SD)	28.1 (28.3)	33.5 (21.2)	68.6 (42.0)	0.03

**Table 4:** Kinetic growth rates. PVE: portal vein embolization; LVD: liver venous deprivation; eLVD: extended LVD. Within the brackets, standard deviations.

Variable	PVE (26)	LVD (10)	eLVD (8)	*p*-Value
KGR—segment 1 (SD)	0.11 (0.11)	0.17 (0.15)	0.27 (0.24)	<0.01
KGR—segments 2+3 (SD)	1.72 (1.72)	1.89 (1.21)	2.89 (1.53)	0.13
KGR—segment 4 (SD)	0.48 (1.07)	0.34 (1.04)	0.23 (0.90)	0.94
KGR—FRL (SD)	2.22 (2.11)	2.43 (3.78)	3.21 (1.37)	0.28
KGR—embolized liver (SD)	−2.22 (2.11)	−2.43 (3.78)	−3.21 (1.37)	0.28

## Data Availability

Dataset available upon request from the authors.
